# Measures of Neighborhood Opportunity and Adherence to Recommended Pediatric Primary Care

**DOI:** 10.1001/jamanetworkopen.2023.30784

**Published:** 2023-08-24

**Authors:** Janani Ramachandran, Stephanie L. Mayne, Mary Kate Kelly, Maura Powell, Katie E. McPeak, George Dalembert, Brian P. Jenssen, Alexander G. Fiks

**Affiliations:** 1 Clinical Futures and Policy Lab, Children’s Hospital of Philadelphia, Philadelphia, Pennsylvania; 2 The Possibilities Project, Children’s Hospital of Philadelphia, Philadelphia, Pennsylvania; 3 The Department of Pediatrics, Perelman School of Medicine, University of Pennsylvania, Philadelphia; 4 Leonard Davis Institute for Health Economics, University of Pennsylvania, Philadelphia

## Abstract

**Importance:**

Neighborhood conditions are known to broadly impact child health. Research to date has not examined the association of the Childhood Opportunity Index (COI), a multidimensional indicator of neighborhood environment conditions, specifically with pediatric primary care outcomes.

**Objective:**

To determine the association of neighborhood opportunity measured by the COI with health metrics commonly captured clinically in pediatric primary care, reflecting both access to preventive care and child well-being.

**Design, Setting, and Participants:**

This cross-sectional observational study used electronic health record data from a large pediatric primary care network in the northeastern US. Participants included patients aged 0 to 19 years who were active in the primary care network between November 2020 and November 2022. Data were analyzed in December 2022.

**Exposure:**

Census tract–level COI overall score (in quintiles).

**Main Outcomes and Measures:**

Outcomes included up-to-date preventive care and immunization status and presence of obesity, adolescent depression and suicidality, and maternal depression and suicidality. Multivariable mixed-effects logistic regressions estimated associations of these outcomes with COI quintiles, adjusted for age, sex, race and ethnicity, and insurance type.

**Results:**

Among 338 277 patients (mean [SD] age, 9.8 [5.9] years; 165 223 female [48.8%]; 158 054 [46.7%] non-Hispanic White, 209 482 [61.9%] commercially insured), 81 739 (24.2%) and 130 361 (38.5%) lived in neighborhoods of very low and very high COI, respectively. Living in very high COI neighborhoods (vs very low COI) was associated with higher odds of being up-to-date on preventive visits (odds ratio [OR], 1.40; 95% CI, 1.32-1.48) and immunizations (OR, 1.77; 95% CI, 1.58-2.00), and with lower odds of obesity (OR, 0.55; 95% CI, 0.52-0.58), adolescent depression (OR, 0.78; 95% CI, 0.72-0.84) and suicidality (OR, 0.79; 95% CI, 0.73-0.85), and maternal depression (OR, 0.78; 95% CI, 0.72-0.86) and suicidality (OR, 0.71; 95% CI, 0.61-0.83).

**Conclusions and Relevance:**

This cross-sectional study of electronic health record data found that neighborhood opportunity was associated with multiple pediatric primary care outcomes. Understanding these associations can help health systems identify neighborhoods that need additional support and advocate for and develop partnerships with community groups to promote child well-being. The findings underscore the importance of improving access to preventive care in low COI communities.

## Key Points

**Question:**

Is neighborhood opportunity, measured with the Childhood Opportunity Index, associated with preventive care access and well-being outcomes in pediatric primary care?

**Findings:**

In this cross-sectional study including 338 277 children aged 0 to 19 years in a large pediatric primary care network, children in very high opportunity neighborhoods were significantly more likely to be up-to-date on preventive visits and vaccinations and were less likely to have obesity or to screen positive for adolescent or maternal depression and suicidality, compared with children in very low opportunity neighborhoods.

**Meaning:**

These findings suggest that neighborhood opportunity is associated with pediatric primary care outcomes related to preventive care access and child well-being, underscoring the importance of improving preventive care resources and access in low-opportunity communities.

## Teaser

This cross-sectional study examines the association of neighborhood opportunity, measured with the Childhood Opportunity Index, with health metrics commonly captured clinically in pediatric primary care, reflecting both access to preventive care and child well-being.

## Introduction

Health inequities among children can have long-term impacts on their health outcomes, educational attainment, and economic success as adults.^1,2^ There is growing awareness that inequities extend beyond individual-level demographic and socioeconomic differences. The built and social environments in which children reside can offer both opportunities and challenges for the well-being of children and play an essential role in their health outcomes across the life course.^3,4,5,6^ Area-level socioeconomic factors, which are shaped by structural and political factors such as discriminatory housing policies and neighborhood disinvestment, have been found to be associated with inequities in pediatric preventive care, which is essential for ensuring healthy child development and early intervention to improve outcomes for several diseases and conditions.^7,8^ The health care system and pediatric clinicians are uniquely positioned to identify and understand such inequities and advocate for efforts to address them at the community level.^9,10^ This role underscores the need for pediatric health systems and clinicians to better understand how neighborhood opportunity and attributes influence the health of patients they serve.

The Childhood Opportunity Index (COI) is a publicly available cumulative indicator of positive and negative attributes of neighborhood conditions and resources that influence healthy child development. The index is built using 29 indicators that span 3 domains: education (eg, number of early child education centers and high school graduation rate), health and environment (eg, walkability and toxic exposures), and social and economic context (eg, employment rate and percentage of households receiving public assistance). The indicators are scaled according to their associations with children’s health and economic outcomes. The COI is a multidimensional measure incorporating data from the US Census Bureau’s American Community Survey and other sources that captures inequities in distribution of opportunity across neighborhoods.^11^ Previous studies^6,12,13,14,15,16^ have explored the associations of COI with acute care use, hospitalizations for ambulatory care sensitive conditions, emergency department visits and readmissions, asthma morbidity, obesity, and cardiometabolic risk in pediatric populations. However, to our knowledge, the association of COI with routine pediatric preventive care access and wellness metrics has not yet been studied in detail. Given the importance of pediatric primary care to establishing healthy trajectories throughout the life course and the longitudinal nature of relationships between primary care clinicians and their patients, a better understanding of the associations of neighborhood opportunity with primary care outcomes is needed.

Our aim was to examine within a large pediatric primary care network the association of neighborhood attributes, as measured by the COI, with preventive care metrics commonly captured clinically in pediatric primary care. We hypothesized that children living in neighborhoods with high COI scores would be more likely than children in neighborhoods with low COI scores to have up-to-date preventive care and immunizations and would be less likely to have obesity or to screen positive for depression or suicidality.

## Methods

### Study Design, Setting, and Population

**Figure 1.**
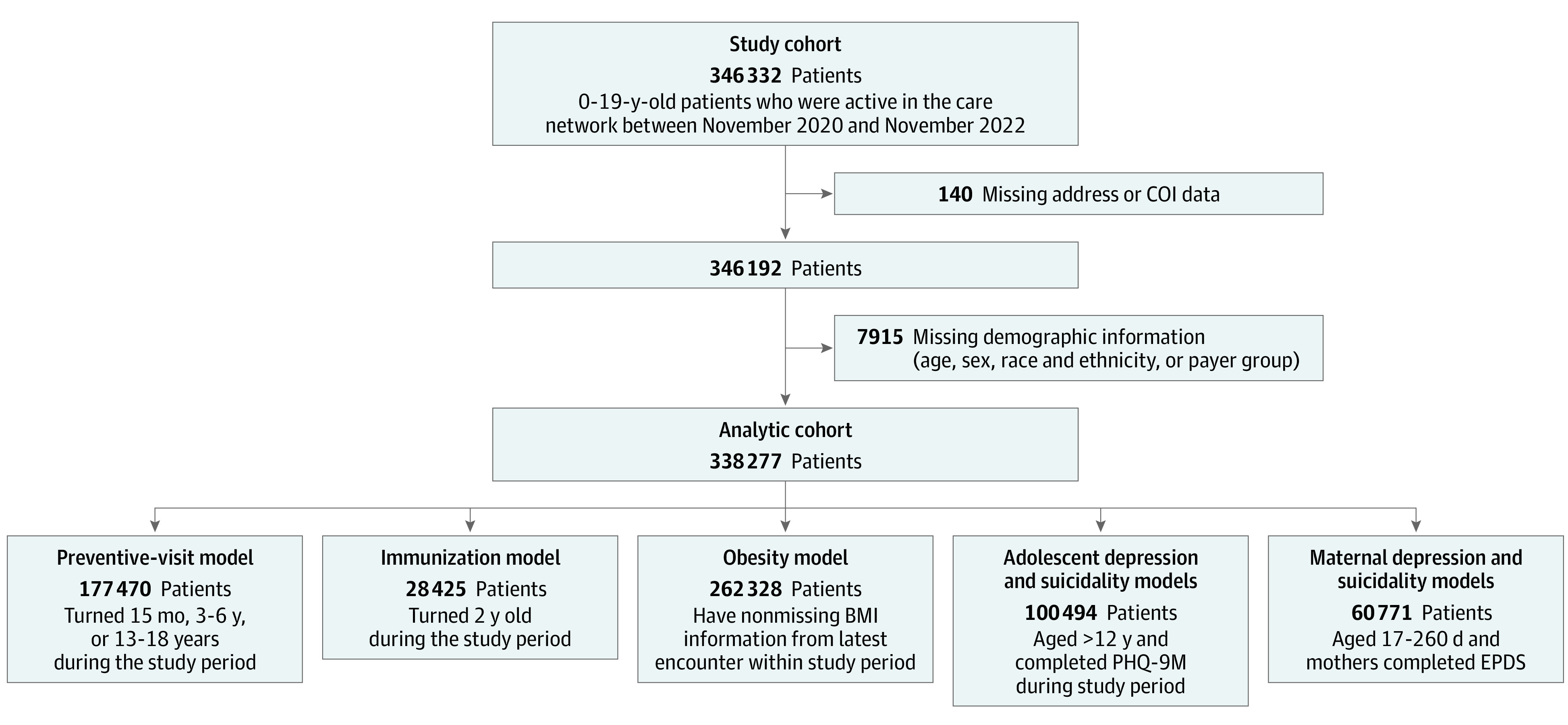
Analytic Cohort Derivation BMI indicates body mass index; COI, Child Opportunity Index; EPDS, Edinburgh Postnatal Depression Scale; and PHQ-9-M, Patient Health Questionnaire-9 Modified for teens.

This retrospective, cross-sectional, observational study used electronic health record (EHR) data from the Children’s Hospital of Philadelphia (CHOP) primary care network. The CHOP network provides care for more than 300 000 patients at 31 pediatric primary care clinics in urban, suburban, and semirural areas of Pennsylvania and New Jersey.^17^ Our study cohort included patients aged 0 to 19 years who were active in the care network between November 2020 and November 2022. At any given time, a patient is considered active if they had at least 1 primary care clinician visit (eg, acute or chronic care or preventive care visits) in the preceding 2 years. To define our analytic cohort, we excluded those with missing address information, COI data, or demographic information (age, sex, race and ethnicity, and payer type). We further derived analytic samples for each outcome of interest as described in Figure 1. The institutional review board of CHOP determined this study to be exempt from their review and waived the requirement for informed consent, because the data were deidentified, in accordance with 45 CFR §46. We followed Strengthening the Reporting of Observational Studies in Epidemiology (STROBE) reporting guidelines for cross-sectional studies for our report.^18^

### Measures

#### Exposure

Our main exposure was the COI version 2.0, which is a publicly available multidimensional indicator of neighborhood conditions and resources conducive to healthy child development.^11^ This cumulative index is calculated at the Census tract level and normalized across the nation, state, or metropolitan area. Patients’ geocoded residential addresses were linked to quintiles of nationally normed overall COI score (ie, very low, Census tracts with scores at or below the 20th percentile; low, tracts above the 20th and at or below the 40th percentile; moderate, tracts above the 40th and at or below the 60th percentile; high, tracts above the 60th and at or below the 80th percentile; and very high, tracts above the 80th percentile), which was used as an ordinal variable in our analyses. COI scores reflect rankings of nearly all Census tracts in the US, such that patients in the very high category were in the top 20% of Census tracts nationwide in terms of neighborhood opportunity. In a secondary analysis, we used the domain-specific opportunity levels for the education, health and environment, and social and economic domains that constitute the COI, as opposed to the overall score.

#### Outcomes

We identified 7 wellness metrics routinely measured in pediatric primary care that incorporate both measures related to preventive care access and child and family wellness throughout different stages of childhood. These outcomes, which are described in detail in eTable 1 in Supplement 1, included adherence to American Academy of Pediatrics’ preventive care guidelines for preventive visit attendance and immunization (measured using Healthcare Effectiveness Data and Information Set measurement definitions),^19,20^ as well as obesity and adolescent and maternal mental health screening results for depressive symptoms and suicide risk. Outcomes were selected to provide a broad view of the association of neighborhood opportunity with child wellness priorities in the primary care setting.

#### Covariates

Sociodemographic variables, including age, sex (male or female), race and ethnicity (Asian, Hispanic, non-Hispanic Black, non-Hispanic White, and other [ie, American Indian or Alaska Native, Native Hawaiian or Other Pacific Islander, multiracial, and unknown or chose not to disclose]), and payer type (publicly insured, privately insured, and other), were identified a priori as potential confounders of the association of neighborhood opportunity with the primary care outcome metrics. These variables were extracted from the EHR. Participant race and ethnicity were identified and classified according to the data available in the EHR. Race and ethnicity were considered as social constructs and markers for potential exposure to structural racism, not as biological variables.

### Statistical Analysis

We used descriptive statistics to describe the demographic characteristics of our cohort and the distribution of COI levels of patients’ residential neighborhoods. Similarly, the proportion of patients who met the outcome criteria in each COI level was explored visually.

We then evaluated the association of COI level with each of our outcome measures by constructing a series of multivariable mixed effects logistic regression models, adjusted for age, sex, race and ethnicity, and payer type. Despite the known associations of COI with race and ethnicity and payer type,^21^ adjusting for them in our models allowed us to determine the association of COI with our outcomes over and above the impact of race and ethnicity and payer type. We included a random intercept for the Census tract of each patient’s address to account for clustering of patients living within the same neighborhood. For each model, odds ratios (ORs) and 95% CIs were obtained, using the very low COI level as the reference group. In a secondary analysis, we examined associations of the 3 COI domains (ie, education, health and environment, and social and economic) with our outcome measures. In addition, we examined unadjusted mixed effects models to evaluate how adjusting for sociodemographic covariates influenced our estimates. Analyses were conducted in December 2022, using R statistical software version 4.1.0 (R Project for Statistical Computing) and Stata statistical software version 16.1 (StataCorp).

## Results

Our analytic cohort of 338 277 children and adolescents had a mean (SD) age of 9.8 (5.9) years, 165 223 (48.8%) were female, 158 054 (46.7%) were identified in the EHR as non-Hispanic White, and 209 482 (61.9%) were commercially insured. With regard to COI level, 81 739 children (24.2%) lived in neighborhoods of very low levels, 36 266 (10.7) lived in neighborhoods of moderate levels, and 130 361 (38.5%) lived in neighborhoods of very high COI levels (Table 1). The distribution of neighborhood COI varied by race and ethnicity and payer type. Non-Hispanic Black patients and publicly insured children were more likely to live in areas of very low and low COI (Table 1). To derive our analytic cohort, we excluded 2.4% of our initial cohort of 346 332 patients aged 0 to 19 years who were active in the network between November 2020 and November 2022 (ie, we excluded 140 patients with missing address or COI data and another 7915 with missing demographic information) (Figure 1).

**Table 1.** Population Characteristics

Characteristic	Participants, No. (%)					
	Overall (N = 338 277)	Very low COI (n = 81 739 [24.2%])	Low COI (n = 25 305 [7.5%])	Moderate COI (n = 36 266 [10.7%])	High COI (n = 64 606 [19.1%])	Very high COI (n = 130 361 [38.5%])
Age, mean (SD), y	9.8 (5.9)	9.6 (5.9)	9.5 (5.9)	9.3 (5.9)	9.7 (6.0)	10.3 (5.9)
Sex						
Female	165 223 (48.8)	40 157 (49.1)	12 351 (48.8)	17 678 (48.8)	31 583 (48.9)	63 454 (48.7)
Male	173 054 (51.2)	41 582 (50.9)	12 954 (51.2)	18 588 (51.3)	33 023 (51.1)	66 907 (51.3)
Race and ethnicity						
Asian	17 204 (5.1)	2387 (2.9)	1409 (5.6)	1286 (3.6)	2671 (4.1)	9451 (7.3)
Hispanic or Latino	30 862 (9.1)	8239 (10.1)	3607 (14.3)	4541 (12.5)	6593 (10.2)	7882 (6.1)
Non-Hispanic Black	90 024 (26.6)	60 050 (73.5)	10 114 (40.0)	7629 (21.0)	6161 (9.5)	6070 (4.7)
Non-Hispanic White	158 054 (46.7)	4290 (5.3)	6773 (26.8)	17 729 (48.9)	40 398 (62.5)	88 864 (68.2)
Other^a^	42 133 (12.5)	6773 (8.3)	3402 (13.4)	5081 (14.0)	8783 (13.6)	18 094 (13.9)
Payer type						
Commercial	209 482 (61.9)	18 777 (23.0)	11 386 (45.0)	21 726 (59.9)	46 753 (72.4)	110 840 (85.0)
Public	123 120 (36.4)	62 109 (76.0)	13 368 (52.8)	13 420 (37.0)	16 646 (25.8)	17 577 (13.5)
Other	5675 (1.7)	853 (1.0)	551 (2.2)	1120 (3.1)	1207 (1.9)	1944 (1.5)

Abbreviation: COI, Child Opportunity Index.

^a^ Other refers to American Indian or Alaskan Native, Native Hawaiian or Other Pacific Islander, multiracial, and unknown or chose not to disclose.

**Figure 2.**
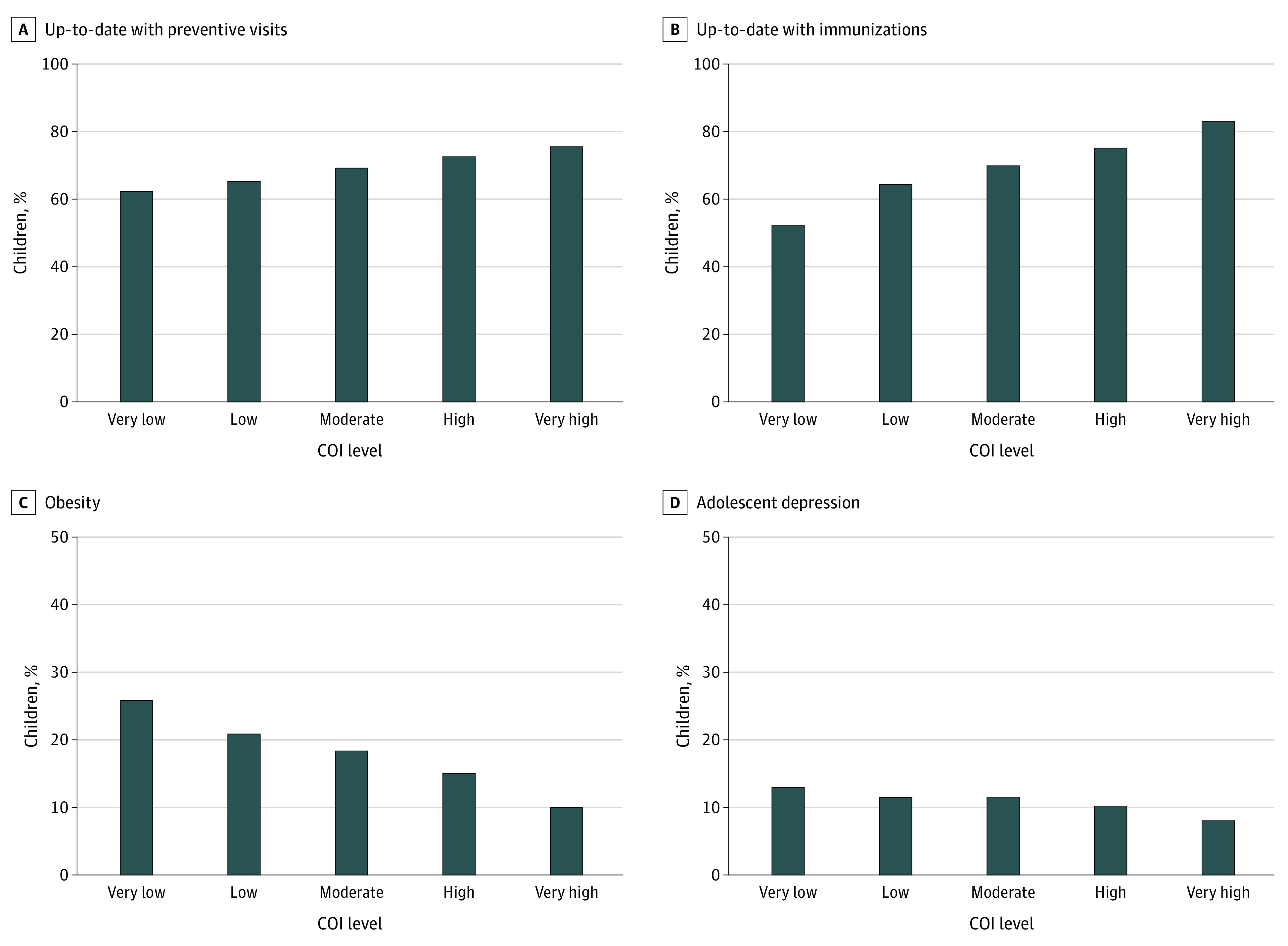
Distribution of 4 Pediatric Primary Care Metrics Across Child Opportunity Index (COI) Levels Graphs show percentages of children with up-to-date preventive visits (A), up-to-date immunizations (B), obesity (C), and depression (D), according to COI level.

In descriptive analyses, the percentage of children with more favorable primary care outcomes increased with increasing neighborhood COI (Figure 2). For example, the percentage of children who were up-to-date on preventive visits was lower in very low opportunity neighborhoods compared with very high opportunity neighborhoods (62.2% [26 935 children] vs 75.5% [53 136 children]), and the percentage of children who were up-to-date on immunizations was lower in very low opportunity neighborhoods, compared with very high opportunity neighborhoods (52.3% [3496 children] vs 83.1% [8915 children]). Patterns were similar for the outcomes related to physical and mental health. The percentage of children meeting criteria for obesity was 25.8% (15 736 children) in very low opportunity neighborhoods but only 10.0% (10 741 children) in very high opportunity neighborhoods, whereas the percentage of adolescents screening positive for moderate-to-severe depressive symptoms was 12.9% (2801 children) in very low opportunity neighborhoods, compared with 8.0% (3549 children) in very high opportunity neighborhoods. Patterns were similar for adolescent suicide risk and maternal depression and suicide risk (eFigure in Supplement 1).

On the basis of our mixed-effects regression models, COI level was found to be associated with all 7 outcomes in the hypothesized direction after adjustment for sociodemographic characteristics available in the EHR (Table 2). Compared with living in very low opportunity neighborhoods, living in very high opportunity neighborhoods was associated with 40% higher odds of being up-to-date on preventive care (OR, 1.40; 95% CI, 1.32-1.48) and 77% higher odds of being up-to-date on immunizations (OR, 1.77; 95% CI, 1.58-2.00). Living in very high opportunity neighborhoods was associated with 45% lower odds of having obesity compared with living in very low opportunity neighborhoods (OR, 0.55; 95% CI, 0.52-0.58). Adolescents in very high vs very low opportunity neighborhoods had 22% lower odds of screening positive for depression (OR, 0.78; 95% CI, 0.72-0.84) and 21% lower odds of screening positive for suicidality (OR, 0.79; 95% CI, 0.73-0.85). Mothers of newborns and infants living in very high opportunity neighborhoods had 22% lower odds of screening positive for maternal depression (OR, 0.78; 95% CI, 0.72-0.86) and 29% lower odds of screening positive for maternal suicidality (OR, 0.71; 95% CI, 0.61-0.83) compared with those from very low opportunity neighborhoods.

**Table 2.** Mixed-Effects Logistic Regression Model: COI Level vs Pediatric Primary Care Health Metrics, Adjusted for Demographic Variables

Health metric	Participants, No.	Adjusted OR (95% CI)^a^			
		Low COI	Moderate COI	High COI	Very high COI
Up-to-date preventive visit	177 470	0.95 (0.89-1.02)	1.09 (1.02-1.17)	1.19 (1.12-1.26)	1.40 (1.32-1.48)
Up-to-date immunization	28 425	1.09 (0.94-1.26)	1.17 (1.02-1.34)	1.22 (1.08-1.38)	1.77 (1.58-2.00)
Obesity	262 328	0.91 (0.85-0.98)	0.90 (0.85-0.96)	0.78 (0.74-0.83)	0.55 (0.52-0.58)
PHQ-9-M adolescent depression screen	100 494	0.94 (0.85-1.04)	1.01 (0.92-1.11)	0.94 (0.87-1.03)	0.78 (0.72-0.84)
PHQ-9-M adolescent suicidality screen	100 494	0.94 (0.86-1.03)	0.92 (0.84-1.00)	0.86 (0.80-0.94)	0.79 (0.73-0.85)
EPDS maternal depression screen	60 771	0.94 (0.85-1.04)	0.96 (0.87-1.05)	0.89 (0.82-0.98)	0.78 (0.72-0.86)
EPDS maternal suicidality screen	60 771	0.96 (0.81-1.14)	0.83 (0.70-0.98)	0.78 (0.67-0.92)	0.71 (0.61-0.83)

Abbreviations: COI, Child Opportunity Index; EPDS, Edinburgh Postnatal Depression Scale; OR, odds ratio; PHQ-9-M, Patient Health Questionnaire-9 Modified for teens.

^a^ Model was adjusted for age, sex, race and ethnicity, and payer type.

When we repeated the mixed-effects regression models for each of the 3 COI domains, the patterns were similar to those for overall COI (eTable 2 in Supplement 1). We also examined the unadjusted model for each outcome and found that adjustment for sociodemographic characteristics slightly attenuated, but did not substantively change the associations (eTable 3 in Supplement 1).

## Discussion

In this observational, cross-sectional analysis of EHR data from a large pediatric primary care network, children who resided in neighborhoods with higher childhood opportunity levels had markedly more favorable outcomes across a range of pediatric primary care access and wellness metrics, including higher odds of up-to-date preventive visits and immunizations, lower odds of obesity, and lower odds of screening positive for adolescent and maternal depression and suicidality. These associations were independent of sociodemographic factors, such as age, sex, race and ethnicity, and payer type, and were consistent across the 3 domains of the COI.

The COI is a metric that is increasingly used for research, community needs assessments, and population health monitoring. An advantage of the COI is that it provides a broad assessment of the neighborhood environment while incorporating features specifically selected because of their importance for healthy child development. Although past studies^13,14,15,22^ have reported that lower neighborhood COI scores were associated with greater pediatric acute and emergency care utilization, hospitalizations for ambulatory care sensitive conditions, asthma hospitalizations, and hospital readmissions, those studies did not examine how neighborhood opportunity relates to pediatric preventive care. Our findings indicate that there are substantial differences in the percentage of children attending recommended preventive visits and receiving immunizations according to neighborhood opportunity, suggesting that many families in low opportunity neighborhoods must overcome substantial barriers to access preventive care. This is consistent with past studies reporting spatial iniquities by neighborhood socioeconomic status in the ratio of pediatric primary care clinicians to pediatric population^23^ and in the completion of recommended preventive services (eg, vaccination and screenings).^8^

Several prior studies^6,16^ using data from longitudinal cohorts have found neighborhood COI to be associated with chronic conditions, such as pediatric obesity and cardiometabolic risk. A large literature base^24,25^ has also established the importance of neighborhood environments more broadly for child physical and mental health. For example, specific neighborhood physical features, such as access to parks, playgrounds, sidewalks, and walking paths, have been found to be associated with childhood obesity, suggesting that better access to these neighborhood attributes may promote physical activity.^26^ Other studies have found lower neighborhood socioeconomic status to be associated with adverse mental health conditions among adolescents,^3,27^ as well as with maternal depression.^28,29^ Living in a disadvantaged neighborhood may increase families’ exposure to a range of stressors, such as poverty, racism, food insecurity, and reduced access to health care, leading to parental stress and negatively impacting mental health outcomes in children.^30^ In contrast, greater neighborhood safety and amenities may foster resilience.^31^ Our study expands on existing research by incorporating a multidimensional measure of neighborhood conditions and by demonstrating the degree to which neighborhood opportunity is associated with aspects of child well-being encountered by clinicians, specifically in the context of pediatric primary care, thereby contributing novel findings to the existing body of knowledge.

The impacts of neighborhood conditions on children may be particularly salient to pediatric primary care clinicians, who often develop long-term relationships with families. Our findings, in conjunction with past research, underscore the potential value for pediatric primary care practices and health systems in using the COI to better understand their patient populations and examine outcomes from an equity-focused perspective. For example, pediatric health systems may use COI data to identify target populations that require additional resources for healthy child development. There may be differences in care-seeking behavior among families in very low COI areas owing to factors such as distrust or the necessity of overcoming structural barriers and managing competing needs, highlighting the need for clinicians to talk to these families and understand their perception of preventive care and barriers and facilitators to seeking it. Health systems might then take actions to reduce barriers to lower COI communities, such as converting triage calls for acute complaints to same-day well-visits and providing vaccinations, developmental screening, and mental health screening at any opportunity of contact. By using the COI in the evaluation of quality improvement efforts, interventions, and innovations, health systems may customize care to promote overall child well-being and ensure equitable allocation of resources to support the health of children across all COI levels. In addition, the COI and its component domains provide a potential road map for identification of strategic community-based partnerships that build on community strengths to advance health equity. For instance, the COI can be used for targeted implementation of medical financial partnerships that are shown to be impactful antipoverty interventions that can be integrated into primary care settings.^32^

### Limitations

Our study is bolstered by a large sample size and a diverse population of children spanning urban, suburban, and semirural settings. However, there are also several important limitations to note. First, the associations observed in our study may not be representative of national associations or those at other hospitals, because the data came entirely from a single health system with clinics mainly in the Philadelphia, Pennsylvania, area and a few New Jersey areas. In addition, the EHR contains limited information on individual, family-level, or environmental features that impact child well-being. Moreover, since our exposure is assigned at the Census tract level, it may not represent the true exposure experienced by individual children or families within each Census tract, possibly leading to misclassification that might attenuate the associations we observed. All our data were obtained from the EHR and patient-reported surveys, both of which could be subject to measurement error. The COI does not include information on access to health care within the neighborhood. Future work could consider leveraging spatial information on access to primary care in conjunction with COI.

## Conclusions

In conclusion, our results indicate that neighborhood attributes, as measured by the COI, are associated with preventive care metrics routinely captured in pediatric primary care, including well-visit and immunization adherence, obesity, and adolescent and maternal mental health. The findings underscore the potential value for pediatric health systems in using data on neighborhood opportunity to identify geographic areas and populations that need additional support, to advocate for neighborhood investments and interventions, to innovate and offer low-barrier preventive care and supportive ancillary services, and to develop partnerships with community groups to promote child well-being. These results also underscore the importance of improving access to pediatric preventive care in low COI communities.

## Notes

Supplement 1. **eTable 1.** Definition of Pediatric Primary Care Metrics **eTable 2.** Mixed Effects Logistic Regression Model: Subdomains of Child Opportunity Index Level vs Pediatric Primary Care Health Metrics, Adjusted for Demographics^a^ **eTable 3.** Unadjusted Mixed Effects Logistic Regression Models: Child Opportunity Index Level vs Pediatric Primary Care Health Metrics **eFigure.** Distribution of Three Pediatric Primary Care Metrics Across COI Levels

Supplement 2.
Data Sharing Statement
