# Supplementary material for: Measures of Neighborhood Opportunity and Adherence to Recommended Pediatric Primary Care
Source: JAMA Netw Open. 2023 Aug 24;6(8):e2330784. doi: 10.1001/jamanetworkopen.2023.30784 (PMC10450570; doi:10.1001/jamanetworkopen.2023.30784)
Supplement: Supplement 1. — eTable 1. Definition of Pediatric Primary Care Metrics eTable 2. Mixed Effects Logistic Regression Model: Subdomains of Child Opportunity Index Level vs Pediatric Primary Care Health Metrics, Adjusted for Demographicsa eTable 3. Unadjusted Mixed Effects Logistic Regression Models: Child Opportunity Index Level vs Pediatric Primary Care Health Metrics eFigure. Distribution of Three Pediatric Primary Care Metrics Across COI Levels [file jamanetwopen-e2330784-s001.pdf]

Supplemental Online Content

Ramachandran J, Mayne SL, Kelly MK, et al. Measures of neighborhood opportunity and adherence to recommended pediatric primary care. *JAMA Netw Open*. 2023;6(8):e2330784. doi:10.1001/jamanetworkopen.2023.30784

eTable 1. Definition of Pediatric Primary Care Metrics
eTable 2. Mixed Effects Logistic Regression Model: Subdomains of Child Opportunity Index Level vs Pediatric Primary Care Health Metrics, Adjusted for Demographics[a]
eTable 3. Unadjusted Mixed Effects Logistic Regression Models: Child Opportunity Index Level vs Pediatric Primary Care Health Metrics
eFigure. Distribution of Three Pediatric Primary Care Metrics Across COI Levels

This supplemental material has been provided by the authors to give readers additional information about their work.

© 2023 Ramachandran J et al. *JAMA Network Open.*

eTable 1: Definition of Pediatric Primary Care Metrics

Outcome	Definition	Model eligibility
Up to Date Preventive Care	HEDIS Metric for child and adolescent preventive visits -<br>- 15 months old: considered up to date if they had ≥6 preventive visits with a primary care physician during their first 15 months of life.<br>- 3-6 years old or 13-18 years old: considered up to date if they had ≥1 preventive visit with a primary care practitioner during the past year;	Patients who turned 15 months, 3-6 years, or 13-18 years old during the study period
Up to Date Immunizations	HEDIS Metric for Immunizations - children who turned 2 years of age during the study period are considered up to date if they had the following vaccines by their 2nd birthday.<br>- 4 diphtheria, tetanus, and acellular pertussis (DTaP)<br>- 3 polio (IPV); one measles, mumps, and rubella (MMR)<br>- 3 Haemophilus influenza type B (HiB)<br>- 3 hepatitis B (HepB),<br>- 1 chickenpox (VZV)<br>- 4 pneumococcal conjugate (PCV)<br>- 1 hepatitis A (HepA)<br>- 2 or 3 rotavirus (RV)<br>- 2 influenza (flu)	Patients who turned 24 months during the study period, had at least one primary care office visit between the age of 15-23 months, and had at least 2 preventive visits in their first year
Obesity	Children were considered obese if their most recently measured body mass index (BMI) was ≥ the 95th percentile for age and sex	Patients with non-missing BMI at their latest primary care visit within the study period
Positive Adolescent Depression Screen	A positive adolescent depression screen was defined as ever having a modified Patient Health Questionnaire (PHQ-9-M) score of 11-27, indicating moderate to severe depression, at any encounter during the study period.	Patients who were aged > 12 years and had completed the PHQ-9-M during a preventive visit within the study period
Positive Adolescent Suicidality Screen	A positive adolescent suicidality screen was defined by ever having a positive endorsement of any of the three suicide risk PHQ-9-M questions: (1) “Thoughts that you would be better off dead, or of hurting yourself in some way?”; (2) “Has there been a time in the past month when you have had serious thoughts about ending your life?"; (3) “Have you ever, in your whole life, tried to kill yourself or made a suicide attempt?”, at any encounter during the study period.	Patients who were aged > 12 years and had completed the PHQ-9-M during a preventive visit within the study period
Positive Maternal Depression Screen	A positive maternal depression screen was defined by ever having an overall score >10 on the Edinburgh Postnatal Depression Scale (EPDS) at any encounter during the study period. The EPDS is administered to mothers during each of their child's preventive care visits between age 17-260 days	Patients who turned 17-260 days within the study period and had at least one preventive visit where their mother completed the EPDS
Positive Maternal Suicidality Screen	A positive maternal suicidality screen was established by ever having a positive response (other than "never”) on item 10 of the EPDS- "The thought of harming myself has occurred to me" at any encounter during the study period. The EPDS is administered to mothers during each of their child's preventive care visits between age 17-260 days	Patients who turned 17-260 days within the study period and had at least one preventive visit where their mother completed the EPDS

* HEDIS- Healthcare Effectiveness Data and Information Set

© 2023 Ramachandran J et al. *JAMA Network Open.*

eTable 2: Mixed Effects Logistic Regression Model: Subdomains of Child Opportunity Index Level vs Pediatric Primary Care Health Metrics, Adjusted for Demographics[a]

COI Level Reference: Very Low	Up to Date Preventive Visit	Up to Date Immunization	Obesity	PHQ9 Depression Screen	PHQ9 Suicidality Screen	Maternal Depression Screen	Maternal Suicidality Screen
**Education Domain**							
Low	1.02 (0.95-1.09)	1.21 (1.06-1.39)	0.91 (0.85-0.97)	0.98 (0.89-1.08)	0.93 (0.85-1.01)	1.00 (0.91-1.10)	0.98 (0.83-1.15)
Moderate	1.05 (0.98-1.12)	0.98 (0.86-1.12)	0.99 (0.92-1.06)	0.97 (0.88-1.06)	0.88 (0.81-0.97)	0.96 (0.87-1.06)	0.87 (0.73-1.04)
High	1.14 (1.07-1.21)	1.21 (1.07-1.37)	0.79 (0.75-0.85)	0.90 (0.83-0.98)	0.84 (0.78-0.91)	0.88 (0.80-0.96)	0.83 (0.70-0.98)
Very High	1.36 (1.29-1.44)	1.72 (1.53-1.92)	0.60 (0.57-0.63)	0.80 (0.74-0.87)	0.81 (0.75-0.87)	0.81 (0.75-0.89)	0.76 (0.65-0.88)
**Health and Environment Domain**							
Low	1.06 (0.99-1.13)	1.08 (0.96-1.22)	0.91 (0.85-0.97)	0.96 (0.89-1.04)	0.93 (0.86-0.99)	0.93 (0.86-1.00)	0.93 (0.82-1.06)
Moderate	1.15 (1.08-1.23)	1.17 (1.03-1.33)	0.78 (0.73-0.83)	0.94 (0.87-1.02)	0.87 (0.80-0.94)	0.90 (0.83-0.98)	0.88 (0.75-1.02)
High	1.24 (1.16-1.32)	1.15 (1.02-1.31)	0.72 (0.67-0.77)	0.91 (0.84-0.99)	0.85 (0.79-0.92)	0.92 (0.84-1.00)	0.81 (0.70-0.95)
Very High	1.30 (1.22-1.39)	1.28 (1.12-1.46)	0.62 (0.58-0.66)	0.79 (0.72-0.86)	0.78 (0.72-0.85)	0.80 (0.73-0.88)	0.71 (0.60-0.85)
**Social and Economic Domain**							
Low	1.01 (0.94-1.09)	1.14 (0.98-1.31)	0.91 (0.84-0.97)	0.96 (0.87-1.06)	0.94 (0.86-1.02)	0.92 (0.84-1.02)	1.05 (0.89-1.23)
Moderate	1.12 (1.05-1.20)	1.20 (1.05-1.36)	0.88 (0.82-0.94)	0.97 (0.89-1.07)	0.90 (0.83-0.98)	0.93 (0.85-1.02)	0.86 (0.73-1.00)
High	1.18 (1.11-1.25)	1.28 (1.14-1.45)	0.77 (0.72-0.81)	0.90 (0.83-0.98)	0.83 (0.76-0.90)	0.88 (0.81-0.97)	0.81 (0.69-0.95)
Very High	1.40 (1.32-1.48)	1.74 (1.54-1.96)	0.56 (0.53-0.59)	0.78 (0.72-0.85)	0.79 (0.73-0.85)	0.78 (0.72-0.86)	0.75 (0.65-0.88)

Abbreviations: COI - Child Opportunity Index, aOR - Adjusted Odds Ratios, PHQ-9-M - Patient Health Questionnaire-9 Modified for teens, EPDS - Edinburgh Postnatal Depression Scale
a – Model adjusted for age, sex, race-ethnicity, payor type

© 2023 Ramachandran J et al. *JAMA Network Open.*

eTable 3: Unadjusted Mixed Effects Logistic Regression Models: Child Opportunity Index Level vs Pediatric Primary Care Health Metrics

COI[ϕ] Level (Reference: Very Low)	n	Low	Moderate	High	Very High
		OR (95% CI)	OR (95% CI)	OR (95% CI)	OR (95% CI)
**Up to Date Preventive Visit**	177,470	1.08 (1.01, 1.16)	1.32 (1.25, 1.40)	1.48 (1.40, 1.56)	1.75 (1.67, 1.83)
**Up to Date Immunization**	28,425	1.64 (1.42, 1.90)	2.22 (1.96, 2.53)	2.74 (2.45, 3.07)	4.70 (4.24, 5.21)
**Obesity**	262,328	0.73 (0.68, 0.79)	0.64 (0.59, 0.68)	0.50 (0.48, 0.53)	0.33 (0.31, 0.34)
**PHQ-9-M Adolescent Depression Screen**	100,494	0.86 (0.78, 0.95)	0.87 (0.79, 0.94)	0.76 (0.71, 0.82)	0.59 (0.55, 0.63)
**PHQ-9-M Adolescent Suicidality Screen**	100,494	0.81 (0.74, 0.89)	0.72 (0.66, 0.78)	0.62 (0.57, 0.66)	0.52 (0.49, 0.55)
**EPDS Maternal Depression Screen**	60,771	0.80 (0.72, 0.88)	0.73 (0.66, 0.80)	0.63 (0.58, 0.68)	0.52 (0.48, 0.55)
**EPDS Maternal Suicidality Screen**	60,771	0.81 (0.68, 0.96)	0.58 (0.49, 0.68)	0.48 (0.42, 0.56)	0.40 (0.35, 0.45)

Abbreviations: COI - Child Opportunity Index, OR - Odds Ratios (Unadjusted), PHQ-9-M - Patient Health Questionnaire-9 Modified for teens, EPDS - Edinburgh Postnatal Depression Scale

© 2023 Ramachandran J et al. *JAMA Network Open.*

eFigure. Distribution of Three Pediatric Primary Care Metrics Across COI Levels

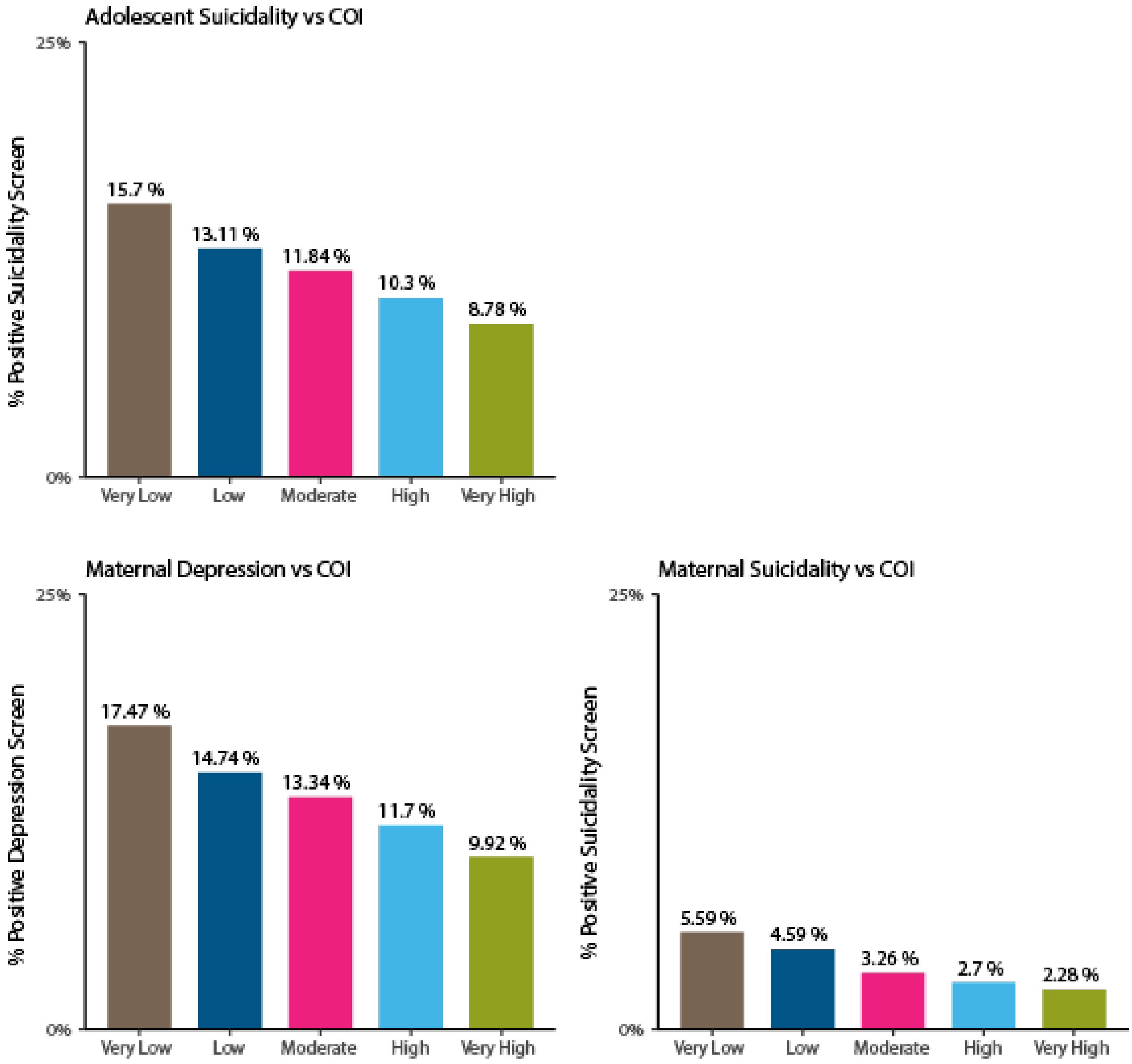

© 2023 Ramachandran J et al. *JAMA Network Open.*
