# Supplementary material for: Measures of Neighborhood Opportunity and Adherence to Recommended Pediatric Primary Care
Source: JAMA Netw Open. 2023 Aug 24;6(8):e2330784. doi: 10.1001/jamanetworkopen.2023.30784 (PMC10450570; doi:10.1001/jamanetworkopen.2023.30784)
Supplement: Supplement 2. — Data Sharing Statement [file jamanetwopen-e2330784-s002.pdf]

Data Sharing Statement

Ramachandran. Measures of Neighborhood Opportunity and Adherence to Recommended Pediatric Primary Care. *JAMA Netw Open*. Published August 24, 2023. doi:10.1001/jamanetworkopen.2023.30784

Data
Data available: No

Additional Information
Explanation for why data not available: The dataset includes individual-level patient health information that will not be shared in order to protect confidentiality.
